# Behavioural difficulties in early childhood and risk of adolescent injury

**DOI:** 10.1136/archdischild-2019-317271

**Published:** 2019-10-30

**Authors:** Amrita Bandyopadhyay, Karen Tingay, Ashley Akbari, Lucy Griffiths, Helen Bedford, Mario Cortina-Borja, Suzanne Walton, Carol Dezateux, Ronan A Lyons, Sinead Brophy

**Affiliations:** 1 National Centre for Population Health and Wellbeing Research, Swansea University Medical School, Swansea, United Kingdom; 2 Administrative Data Research UK, Swansea University Medical School, Swansea, United Kingdom; 3 Office for National Statistics, Cardiff Road, Newport, Wales, UK; 4 Health Data Research UK, Swansea University Medical School, Swansea, United Kingdom; 5 Life Course Epidemiology and Biostatistics, UCL Great Ormond Street Institute of Child Health, UCL, London, UK; 6 Clinical Epidemiology, Nutrition and Biostatistics, UCL Great Ormond Street Institute of Child Health, London, UK; 7 Centre for Primary Care and Public Health, Queen Mary University of London, London, UK

**Keywords:** longitudinal data linkage, routine data, Strengths and Difficulties Questionnaire, hospital admission, A&E attendance

## Abstract

**Objective:**

To evaluate long-term associations between early childhood hyperactivity and conduct problems (CP), measured using Strengths and Difficulties Questionnaire (SDQ) and risk of injury in early adolescence.

**Design:**

Data linkage between a longitudinal birth cohort and routinely collected electronic health records.

**Setting:**

Consenting Millennium Cohort Study (MCS) participants residing in Wales and Scotland.

**Patients:**

3119 children who participated in the age 5 MCS interview.

**Main outcome measures:**

Children with parent-reported SDQ scores were linked with hospital admission and Accident & Emergency (A&E) department records for injuries between ages 9 and 14 years. Negative binomial regression models adjusting for number of people in the household, lone parent, residential area, household poverty, maternal age and academic qualification, child sex, physical activity level and country of interview were fitted in the models.

**Results:**

46% of children attended A&E or were admitted to hospital for injury, and 11% had high/abnormal scores for hyperactivity and CP. High/abnormal or borderline hyperactivity were not significantly associated with risk of injury, incidence rate ratio (IRR) with 95% CI of the high/abnormal and borderline were 0.92 (95% CI 0.74 to 1.14) and 1.16 (95% CI 0.88 to 1.52), respectively. Children with borderline CP had higher injury rates compared with those without CP (IRR 1.31, 95% CI 1.09 to 1.57).

**Conclusions:**

Children with high/abnormal hyperactivity or CP scores were not at increased risk of injury; however, those with borderline CP had higher injury rates. Further research is needed to understand if those with difficulties receive treatment and support, which may reduce the likelihood of injuries.

**What is already known on this topic?**

- Childhood injury is a leading cause of avoidable mortality and morbidity and disproportionately affects children from disadvantaged backgrounds.
- Children with behavioural difficulties have an increased immediate injury risk.

**What this study adds?**

- This longitudinal data linkage study found no association between high levels of behavioural difficulties in early childhood and risk of injury in early adolescence.
- Children with borderline conduct problems are at higher long-term injury risk.
- Further work is needed to delineate the persistence of behavioural difficulties through childhood and their relation to support received and subsequent injury risk.

## Introduction

Injury is the leading cause of mortality and ill-health in adolescence.^1 2^ It is more common among children from disadvantaged backgrounds and hence contributes to health inequalities.^3^ Every year around two million of the overall injury-related visits to Accident & Emergency (A&E) departments involve children and young people in the UK.^4^ Investigating modifiable factors associated with increased risk of injury is important to inform appropriate prevention strategies.

Boys, and children involved in higher level of physical activity, in families with a higher poverty level and of younger mothers are at increased risk of childhood injury.^5–8^ Attention-deficit hyperactive disorder (ADHD) is one of the most common neuropsychiatric disorders of childhood, with an incidence of 3% to 7% in school-aged children.^9^ It is characterised by a higher level of hyperactivity, impulsivity and inattention; children with ADHD are known to be accident-prone with almost a twofold increased risk of injury than children without ADHD.^10^ ADHD is diagnosed according to the core symptoms appearing in the DSM-5 (Diagnostic and Statistical Manual of Mental Disorders).^11^ Treatment comprises medication such as stimulants or selective norepinephrine reuptake inhibitors such as atomoxetine along with parent training programmes and cognitive–behavioural therapy. Children with ADHD are often unable to estimate the risks associated with their activities, elevating their risk of injuries. Previous research has suggested that hyperactivity measured by the Strengths and Difficulties Questionnaire (SDQ) is associated with an increased risk of unintentional injury.^12 13^ Conduct problems (CP) is another externalising behavioural difficulty, which can be conceptualised as antisocial, defiant, aggressive and criminal behavioural pattern in children, which can elevate their injury risk.^11 12^ The overall lifetime prevalence of CP is estimated at 9.5%,^14^ but in school-aged children it is around 3%^15^ and it is twice as prevalent in boys than girls. Treatment for CP is focused on parent training, family therapy, school behavioural supports with medication prescribed only if treating coexisting ADHD. Previous research suggests that CP is not associated with injury risk after adjusting for ADHD.^16^

To date, the risk association between behavioural difficulty and injury has only been investigated over relatively short periods of follow-up^13 17–20^ and it is unclear whether reported associations persist. Previous research also largely relied on self/proxy reports of injury, rather than objective records of injury such as A&E, general practice (GP) or hospital admission (HA) records,^13 17–20^ which might be affected by recall bias.^21^ We aimed to explore the relationship between early childhood behaviour (as measured by the SDQ), at the age of 5 years, and the risk of injury in early adolescence to identify children who might be at increased risk of future injury.

## Methods

### Sample

Data were analysed from the Millennium Cohort Study (MCS), a UK-wide nationally representative longitudinal birth cohort of 18 819 singleton children born between September 2000 and January 2002.^22^ Parents were first interviewed in the home when their child was around 9 months old, with subsequent interviews held at 3, 5, 7 and 11 years of age. At age 7, parents gave written consent to link MCS records to their child’s routine health records up to their 14th birthday. There were 13 681 singleton children who participated in the age 7 survey, 1951 and 1598 of whom were living in Wales and Scotland, respectively, at the time of interview. Consent for health record linkage was obtained for 3304 singleton children (1839 from Wales and 1465 from Scotland).

### Linked cohort

**Figure 1:**
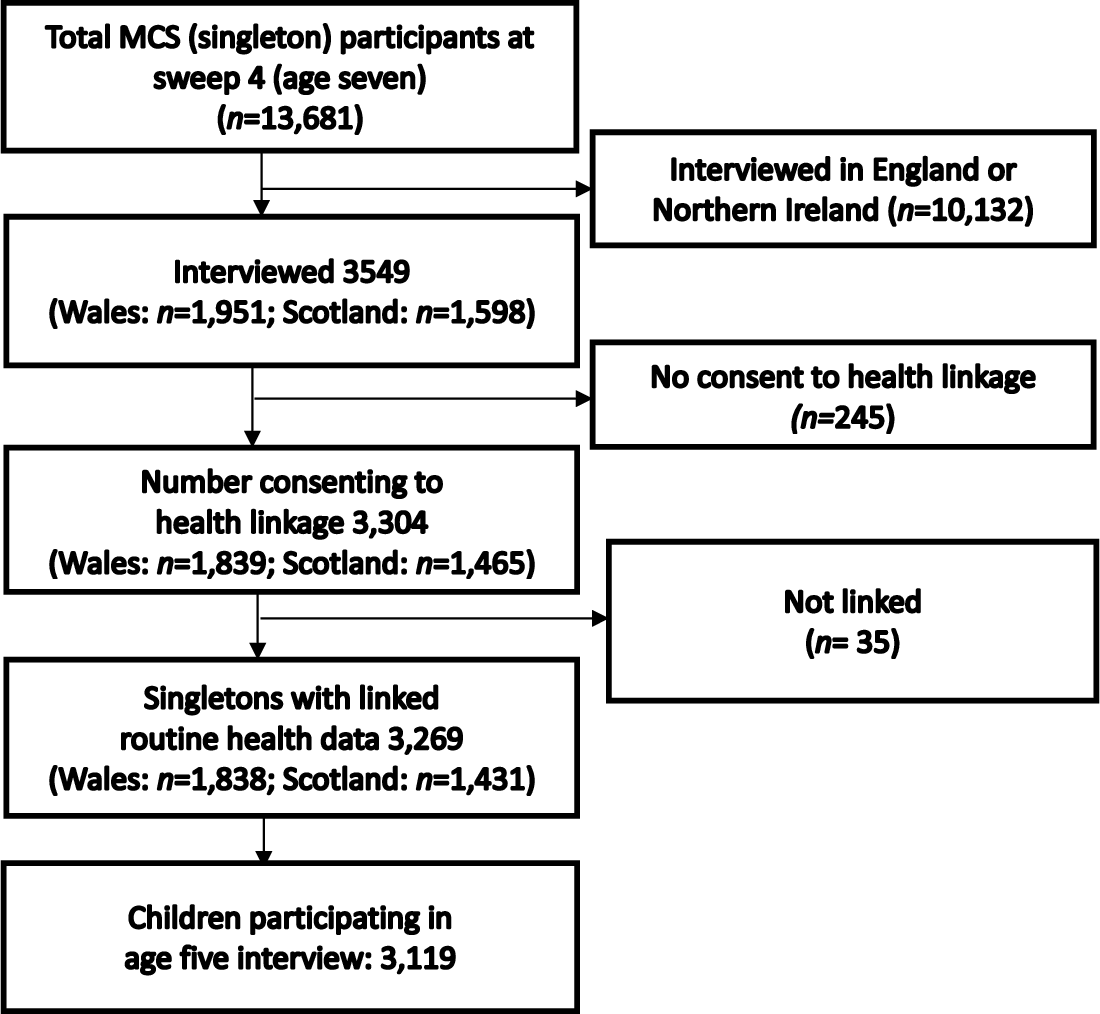
Flow chart of participants. MCS, Millennium Cohort Study.

Consented singleton children who participated in the third MCS survey at age 5 years were linked anonymously to their HAs and the A&E department attendances occurring between the ages of 9 and 14 years. The anonymised MCS birth cohort was linked to routinely collected health datasets stored within the privacy protecting Secure Anonymised Information Linkage (SAIL) Databank.^23 24^ The linkage procedure has been described in detail elsewhere.^25^ Of 3304 consented singleton children, 3269 were linked with their electronic health records (EHRs), of whom 1838 were from Wales and 1431 from Scotland. The study population comprised 3119 children who participated in the age 5 survey in Wales and Scotland and were linked to their health data (figure 1).

### Exposure variables

Child behavioural difficulties were assessed by parent-reported SDQ when children were 5 years old. The SDQ is an internationally validated and widely used screening tool to measure child and adolescent behavioural and emotional difficulties.^26 27^ In this study, two SDQ subscales were examined: hyperactivity and inattention (restless/overactive, constantly fidgeting, easily distracted, cannot stop and think before acting, lack of attention span) and CP (often has temper, tantrums, disobedience, fights with/bullies other children, often lies or cheats, steals). Each subscale has scores between 0 and 10 with higher scores indicating greater level of difficulty. In this study, we used Goodman’s proposed categorisation to assign children into one of three groups: ‘normal’ (hyperactivity: 0–5; CP: 0–2;), ‘borderline’ (hyperactivity: 6; CP: 3) or ‘high/abnormal’ (hyperactivity: 7–10; CP: 4–10).^28^ In this study, analyses were performed for all children who had valid hyperactivity (n=3095) and CP (n=3100) scores.

### Outcome variable

The outcome variable was the frequency of injury-related HAs and/or A&E attendances occurring between the age of 9 and 14 years. We identified HA from the Patient Episode Database for Wales (PEDW) and Scottish Morbidity Records dataset for Wales and Scotland, respectively. An injury-related inpatient HA was identified from emergency admissions with an injury diagnosis International Classification of Diseases, version 10 (ICD-10) code appearing in the first diagnostic position, indicating that injury was the primary cause of admission.^29^ ICD-10 codes are provided in online supplementary appendix table A1.

10.1136/archdischild-2019-317271.supp1 Supplementary data

We identified A&E attendances from the Emergency Department dataset (EDDS) and the Scottish Accident and Emergency version 2 (A&E2) dataset for Wales and Scotland respectively. Data were collected in EDDS from 2009 and in A&E2 from 2007 onwards. To harmonise the data from both countries, the study focused on A&E attendances recorded from 2009. In addition to the ICD-10 codes, the alphanumeric treatment and diagnosis codes which map to an injury type have been included in the study^30 31^ to identify the A&E attendances (refer to online supplementary appendix tables A2 and A3). In this study, simultaneous presence of a patient in both harmonised HA and A&E datasets on the same day, which presumably indicates the transfer from A&E to hospital, has been considered as one record. The data were available as aggregated frequencies of injury-related HAs or A&E attendance per child between ages 9 and 14.

10.1136/archdischild-2019-317271.supp2 Supplementary data

10.1136/archdischild-2019-317271.supp3 Supplementary data

### Covariates

The covariates considered here as confounders in the associations between child behavioural difficulties and injury risk include child’s sex, maternal age at child’s birth, maternal highest academic and vocational qualification (derived by National Vocational Qualifications standard), lone parent carer, household poverty (household income less than 60% of national median using modified Organisation for Economic Co-operation and Development scale), number of people in the household, residential area (using 2005 Rural/Urban Area Classification) and the child’s physical activity level (number of days per week they were involved in sports/exercise). With the exception of sex and maternal age at child’s birth (which were collected from age 9 months MCS interview data), these covariates were derived from age 5 MCS interview data.

### Statistical analysis

We used negative binomial regression models, as the outcome variable was overdispersed, as indicated by the conditional variances being at least twice the conditional means across all categories of both hyperactivity and CP (see online supplementary appendices 4 and 5). For each SDQ scale, ‘normal’ was the reference group compared with the ‘borderline’ and ‘high/abnormal’ groups in each model. We adjusted these models for the covariates described in the previous section (model 1) and considered models with hyperactivity and CP as main exposure variables as well as a covariate (model 2). We did not contrast these two models in terms of goodness-of-fit criteria, as we were interested principally in the results from model 2, whereas model 1 offered a comparator in terms of the possible changes in the risk measure when the other exposure variable was included as a covariate. Sex-stratified models (models 3 and 4) for each exposure variable were also fitted due to higher levels of hyperactivity and CP in boys and higher levels of injury in boys in general. The models’ results were parameterised using incidence rate ratios (IRRs) with 95% CIs. Data preparation including extraction, cleaning and linkage were performed in Structured Query Language on IBM DB2 platform, with all statistical analyses performed in R version 3.3.2.^32^ All the models’ parameters were estimated adjusting for survey and non-response consent weights to account for oversampling, attrition between consent and non-consent to data linkage in the MCS. Survey and non-response consent weights were obtained via predicted probabilities obtained from logistic regression models taking the stratified cluster sampling design into account and adjusting for low representation of children from Wales, Scotland and Northern Ireland, disadvantaged areas and areas with high proportions of ethnic minority groups. The detailed methodological approach to derive the weight variable has been explained elsewhere.^33^

10.1136/archdischild-2019-317271.supp5 Supplementary data

10.1136/archdischild-2019-317271.supp4 Supplementary data

## Results

Table 1 shows the demographic characteristics of the 3119 consented singleton children who participated in the third survey.

**Table 1:** Characteristics of all children with linked EHRs in the study population

	n=3119* (weighted %)†
Hyperactivity	
Normal	2563 (82.8)
Borderline	204 (6.5)
High/abnormal	328 (10.6)
Conduct Problems	
Normal	2404 (76.1)
Borderline	391 (13.4)
High/abnormal	305 (10.5)
Sex	
Boy	1604 (51.3)
Girl	1515 (48.7)
No of people in the household	
2	169 (6.4)
3	564 (18.3)
4	1420 (43.9)
five or more	966 (31.4)
Lone parent	
Lone	586 (20.4)
Non-lone	2533 (79.6)
Residential area	
Rural	756 (26.1)
Urban	2360 (73.9)
Household poverty	
OECD 60% median or above	2213 (69.1)
Below OECD 60% median	894 (30.9)
Maternal education	
Degree	635 (19.3)
Diplomas in Higher Education	344 (9.9)
Advanced/Advanced Subsidiary/Subsidiary levels	401 (14.4)
Ordinary level/General Certificate of Secondary Education	1248 (40.0)
Other	77 (2.4)
None	407 (14.0)
Physical activity level	
3 or more days a week	411 (14.6)
2 days a week	581 (18.5)
1 day a week	872 (27.7)
Less often or not at all	1246 (39.1)
Country	
Wales	1750 (36.3)
Scotland	1369 (63.7)
	**Median (IQR**)
Maternal age at birth (years)	29 (24–33)

*Missing data: hyperactivity (24, unweighted %=0.8); CP (19, unweighted %=0.6); living area (less than 5); poverty indicator (12, unweighted %=0.4); maternal education (7, unweighted %=0.2); physical activity level (9, unweighted %=0.3); maternal age at birth (less than 5).

†Weighting was based on 1 minus the probability of non-consent, multiplied by the age 7 survey weights for data linkage and scaled to the sum of the number of consenting children.

CP, conduct problem; EHR, electronic health record; OECD, Organisation for Economic Co-operation and Development.

Between ages 9 and 14 years, around 46% children had at least one HA or A&E attendance for injury (table 2). There were 2904 records of injury of which 6% were HAs and 94% were A&E attendances.

**Table 2:** Number of children with injury records and number of injury-related hospital admissions or A&E attendance records combined by country

Country	No of children with injury (out of n=3119)	Injury admissions/attendances between 9 and 14 years
Wales	866	1782 (HA=104; A&E=1678)
Scotland	562	1122 (HA=70; A&E=1052)
Total	1428 (45.78%)	2904 (HA=174; A&E=2730)

A&E, Accident & Emergency; HA, hospital admission.

### Associations between SDQ scores and the risk of injury

Hyperactivity at age 5 was not associated with a higher risk of injury in adolescence after adjusting for confounders (table 3). Unadjusted borderline hyperactivity, rather than high/abnormal, were associated with injury (IRR=1.34, 95% CI 1.01 to 1.77). However, this risk attenuated after adjusting for confounding variables, and this effect was similar when CP was included as a covariate.

**Table 3:** IRRs for association between hyperactivity and CPs at age 5 and subsequent hospital admissions or A&E attendances for injury between ages 9 and 14 years

	Unadjusted		Adjusted (model 1)*		Adjusted (model 2)†	
	IRR (95% CI)	P value	IRR (95% CI)	P value	IRR (95% CI)	P value
Hyperactivity						
Normal	1		1		1	
Borderline	1.34 (1.01 to 1.77)	0.040	1.21 (0.92 to 1.59)	0.169	1.16 (0.88 to 1.52)	0.299
High	1.10 (0.91 to 1.34)	0.321	0.98 (0.81 to 1.19)	0.837	0.92 (0.74 to 1.14)	0.435
CPs						
Normal	1		1		1	
Borderline	1.38 (1.16 to 1.65)	<0.001	1.31 (1.09 to 1.59)	0.003	1.31 (1.10 to 1.57)	0.003
High	1.26 (1.02 to 1.56)	0.029	1.12 (0.90 to 1.39)	0.307	1.12 (0.89 to 1.42)	0.326

*Model 1: Adjusted for child’s sex, number of people in household, lone parent, residential area, household poverty, maternal education, physical activity level, maternal age at child’s birth and country of the respondents.

†Model 2: Adjusted for child’s sex, number of people in household, lone parent, residential area, household poverty, maternal education, physical activity level, maternal age at child’s birth, country of the respondents, CP (in case of hyperactivity) and hyperactivity (in case of CP).

A&E, Accident & Emergency; CP, conduct problem; IRR, incidence rate ratio.

High/abnormal CP at age 5 were not associated with higher risk of injury in adolescence once adjustment was made for confounding factors (sex, demographic, family factors and socioeconomic variables). However, borderline CP were associated with a higher rate of injury, even when adjusting for confounding factors and hyperactivity (IRR 1.31, 95% CI 1.10 to 1.57). This was especially true for girls with borderline CP (IRR 1.37, 95% CI 1.04 to 1.8), see table 4.

**Table 4:** IRRs for association between hyperactivity and conduct problems at age 5 and subsequent hospital admissions or A&E attendances for injury between ages 9 and 14 years: sex-stratified models

	Boys				Girls			
	Adjusted (model 3)*		Adjusted (model 4)†		Adjusted (model 3)*		Adjusted (model 4)†	
	IRR (95% CI)	P value	IRR (95% CI)	P value	IRR (95% CI)	P value	IRR (95% CI)	P value
Hyperactivity								
Normal	1		1		1		1	
Borderline	1.05 (0.72 to 1.53)	0.810	1.00 (0.70 to 1.44)	0.982	1.42 (0.91 to 2.21)	0.119	1.33 (0.86 to 2.06)	0.194
High	0.98 (0.77 to 1.25)	0.888	0.93 (0.72 to 1.20)	0.571	0.99 (0.70 to 1.39)	0.937	0.91 (0.62 to 1.33)	0.618
Conduct problems								
Normal	1		1		1		1	
Borderline	1.27 (0.97 to 1.66)	0.088	1.28 (0.98 to 1.67)	0.068	1.38 (1.06 to 1.81)	0.017	1.37 (1.04 to 1.80)	0.023
High	1.07 (0.83 to 1.38)	0.602	1.10 (0.85 to 1.42)	0.465	1.24 (0.81 to 1.91)	0.318	1.20 (0.78 to 1.85)	0.396

*Model 3:Adjusted for number of people in the household, lone parent, residential area, household poverty, maternal education, physical activity level, maternal age at child’s birth and country of the respondents.

†Model 4: Adjusted for number of people in the household, lone parent, residential area, household poverty, maternal education, physical activity level, maternal age at child’s birth and country of the respondents and also adjusted for conduct problems (in case of hyperactivity) and hyperactivity (in case of conduct problems).

A&E, Accident & Emergency; IRR, incidence rate ratio.

## Discussion

Our findings suggest that children with high hyperactivity and CP do not have an increased risk of subsequent injury in early adolescence. However, borderline CP, especially in girls, were associated with a greater risk of injury in adolescence. These findings differ from existing studies,^12 13 19^ which suggested a significant association between hyperactivity, CP and risk of injury. It is possible that these difficulties do not persist throughout childhood, reflecting either spontaneous resolution or, potentially, the effects of interventions, which include pharmacological or cognitive–behavioural treatments^34^ and this may reduce the subsequent injury risk over time. On the other hand, children with borderline disruptive behaviours may not get equivalent family support, parent training/family therapy and school behavioural support. Clinical guidelines for children with behavioural problems have been shown to be inconsistent and difficult to implement due to high caseloads, time pressure and lack of specialised staff.^35^ This may result in children with borderline problems not receiving adequate support with potential implications for persistence or worsening of their problems.

### Strengths and limitations

A strength of the current study is the longitudinal linkage between routinely collected EHRs with longitudinal survey data^25^ which allowed us to examine prospectively recorded injury occurring between 4 and 9 years following assessment of behavioural difficulties. In the current study, we used objective measures of injury and were able to include a longer period of follow-up of the participants, thereby overcoming some of the limitations of previous studies.

However, our study does rely on parent-reported SDQ data. It is possible that parent-reported behaviours may reflect parental perceptions and their ability to cope with child behaviours and so may be subject to bias, for example, parents who are less able to cope or mothers with psychological distress may overestimate their child’s behavioural difficulties.^20 36^ This may explain why some high/abnormal children were not at risk of injury as their difficulties could have been overestimated by parents. Comparison with teacher assessment would have helped to validate the exposure SDQ measures.

Finally, due to the unavailability of the A&E data prior to 2009, the study included the injury records of participants’ between the ages of 9 and 14. Intervention before age 9 due to high rate of injury can reduce the subsequent injury risk, hence data prior to 2009 would have enabled us to investigate the mediating effect of early injury history on the injury risk in adolescence. In this study, GP data were not included; as we did not have GP data for participants from Scotland, we considered that A&E attendances were less likely to include ‘worried well’ parents. This study considered the first diagnostic code within PEDW to identify the cause of admission and disregarded the secondary or other diagnostic positions to avoid the inclusion of pre-existing comorbidities. This might underestimate some injuries, which were wrongly placed when recorded. In this study, the data were available at an aggregated level per child and the time to injury was not taken into consideration within the current study design. Hence we were not able to distinguish between children with many injuries over a short time frame compared with those who have had them over a longer period. In this study, the missing data for the behavioural difficulties were excluded from the analysis; however, due to the small amount of the missing data, the impact of this exclusion is negligible. Additionally, the study does not capture any injuries that do not result in any healthcare contacts; therefore, the observed association between the behavioural difficulties and injury may have been underestimated.

## Conclusion

We found no evidence that high/abnormal levels of hyperactivity or CP at school entry are associated with injury risk in later childhood/early adolescence. There is some evidence that borderline CP is associated with injury risk, especially for girls. Children identified as having significant hyperactivity or CP might have received early support or treatment mitigating their risk of long-term injury in adolescence. However, those with borderline problems may also be at risk but do not receive necessary support thus maintaining their risk of injuries. Further research is needed to clarify the relation of interventions to behavioural trajectories in early childhood and to investigate whether this modifies future injury risk.
