# Supplementary material for: Behavioural difficulties in early childhood and risk of adolescent injury
Source: Arch Dis Child. 2019 Oct 30;105(3):282–7. doi: 10.1136/archdischild-2019-317271 (PMC7041499; doi:10.1136/archdischild-2019-317271)
Supplement: Supplementary data [file archdischild-2019-317271supp001.pdf]

Appendix A1: ICD10 injury codes

Chapter XIX Injury, poisoning and certain other consequences of external causes	
Injuries to the head	S00-S09
Injuries to the neck	S10-S19
Injuries to the thorax	S20-S29
Injuries to the abdomen, lower back, lumbar spine and pelvis	S30-S39
Injuries to the shoulder and upper arm	S40-S49
Injuries to the elbow and forearm	S50-S59
Injuries to the wrist and hand	S60-S69
Injuries to the hip and thigh	S70-S79
Injuries to the knee and lower leg	S80-S89
Injuries to the ankle and foot	S90-S99
Injuries involving multiple body regions	T00-T07
Injuries to unspecified part of trunk, limb or body region	T08-T14
Effects of foreign body entering through natural orifice	T15-T19
Burns and corrosions	T20-T32
Frostbite	T33-T35
Poisoning by drugs, medicaments and biological substances	T36-T50
Toxic effects of substances chiefly nonmedicinal as to source	T51-T65
Effects of air pressure and water pressure	T704, T708, T709
Asphyxiation	T71
Maltreatment syndromes	T741-T749
Effects of other external causes	T751, T754, T79
**Chapter XX External causes of morbidity and mortality**	
Pedestrian injured in transport accident	V01-V09
Pedal cyclist injured in transport accident	V10-V19
Motorcycle rider injured in transport accident	V20-V29
Occupant of three-wheeled motor vehicle injured in transport accident	V30-V39
Car occupant injured in transport accident	V40-V49
Occupant of pick-up truck or van injured in transport accident	V50-V59
Occupant of heavy transport vehicle injured in transport accident	V60-V69
Bus occupant injured in transport accident	V70-V79
Other land transport accidents	V80-V89
Water transport accidents	V90-V94
Air and space transport accidents	V95-V97
Other and unspecified transport accidents	V98-V99
Falls	W00-W19
Exposure to inanimate mechanical forces	W20-W49
Exposure to animate mechanical forces	W50-W64

Accidental drowning and submersion	W65-W74
Other accidental threats to breathing	W75-W84
Exposure to electric current, radiation and extreme ambient air temperature and pressure	W85-W99
Exposure to smoke, fire and flames	X00-X09
Contact with heat and hot substances	W00-W19
Contact with venomous animals and plants	W20-W49
Exposure to forces of nature	W50-W64
Accidental poisoning by and exposure to noxious substances	W65-W74
Intentional self-harm	X60-X84
Assault	X85-Y09
Event of undetermined intent	Y10-Y34
Legal intervention and operations of war	Y35-Y36
Evidence of alcohol involvement determined by blood alcohol level / level of intoxication	Y90-Y91
**Chapter V Mental and behavioural disorders**	
Mental and behavioural disorders due to psychoactive substance use – acute intoxication	F100, F110, F120, F130, F140, F150, F160, F170, F180, F190
