# Supplementary material for: Behavioural difficulties in early childhood and risk of adolescent injury
Source: Arch Dis Child. 2019 Oct 30;105(3):282–7. doi: 10.1136/archdischild-2019-317271 (PMC7041499; doi:10.1136/archdischild-2019-317271)
Supplement: Supplementary data [file archdischild-2019-317271supp002.pdf]

Appendix A2: Welsh A&E (EDDS) injury codes

Variable name	Value	Meaning
		**Wound**
Diagnosis code	03A	Laceration
	03B	Contusion
	03C	Abrasion
	03D	Soft tissue inflammation
	03Z	Wound, other or unspecified
		**Head Injury**
	02A	Glasgow Coma Score 35
	02B	Glasgow Coma Score <35
	02C	Dental Injury
	02Z	Head Injury, other or unspecified
		**Fracture**
	03A	Open Fracture
	03B	Closed Fracture
	03C	Fracture Dislocation
	03Z	Fracture, other or unspecified
		**Joint Injury**
	04A	Sprain
	04B	Dislocation
	04C	Subluxation
	04Z	Joint Injury, other or unspecified
		Amputation
	05Z	Amputation, other or unspecified
		**Soft Tissue Injury**
	06A	Muscle Injury
	06B	Tendon Injury
	06C	Nerve Injury
	06D	Visceral Injury
	06E	Vascular Injury
	06Z	Soft Tissue Injury, other or unspecified
		**Burns, Scalds and Thermal Conditions**
	07A	Electric
	07B	Chemical
	07C	Radiation
	07D	Scald
	07E	Sunburn
	07F	Hyperthermia
	07G	Hypothermia
	07H	Frostbite
	07Z	Burns, Scalds and Thermal Conditions, other or unspecified
		**Foreign Body**
	08A	Ingested Foreign Body
	08Z	Foreign Body, other or unspecified

		Puncture Wounds
	09A	Needle Stick Injury
	09B	Human Bite
	09C	Animal Bite
	09D	Insect Bite or Sting
	09Z	Puncture Wounds, other or unspecified
		**Poisoning or Overdose**
	30A	Alcohol
	30B	Prescribed Drug
	30C	Non-prescribed/purchased drug
	30D	Illicit Drug
	30Z	Poisoning or Overdose, other or unspecified
		**Drowning**
	33A	Near Drowning
	33Z	Drowning, other or unspecified
Diagnosis code		All ICD 10 mentioned in Appendix 1
Treatment code		**Wound Closure**
	03Z	Wound Closure
		Removal Foreign Body
	04Z	Removal Foreign Body
		Manipulation
	06Z	Manipulation
