# Supplementary material for: Behavioural difficulties in early childhood and risk of adolescent injury
Source: Arch Dis Child. 2019 Oct 30;105(3):282–7. doi: 10.1136/archdischild-2019-317271 (PMC7041499; doi:10.1136/archdischild-2019-317271)
Supplement: Supplementary data [file archdischild-2019-317271supp003.pdf]

Appendix A3: Scottish A&E (A&E2) injury codes

Variable name	Value	Meaning
Nature of injury	01	**Wound**
	01A	Abrasion
	01B	Contusion includes bruise, haematoma
	01C	Blisters
	01D	Laceration
	01E	Incision
	01F	Needlestick
	01G	Bite animal
	01H	Bite human
	01J	Bite/sting insect
	01K	Flap laceration
	01L	Degloving wound
	01M	Penetrating wound
	01N	Skin avulsion
	01Z	Other
	02	**Bone and joint injury**
	02A	Closed fracture
	02B	Open fracture
	02C	Dislocation of joint
	02D	Fracture dislocation
	03	**Soft tissue injury**
	03A	Crush injury
	03B	Ligament avulsion
	03C	Ligament tear
	03D	Ligament rupture
	03E	Ligament strain
	03F	Muscle avulsion
	03G	Muscle tear
	03H	Muscle rupture
	03J	Muscle strain
	03K	Tendon dislocation
	03L	Tendon rupture
	03M	Tendon subluxation
	03N	Tendon strain
	03P	Tendon division
	03Q	Neuropraxia
	03R	Nerve compression
	03S	Complete transection of nerve
	03T	Partial division of nerve
	03U	Nerve contusion
	03V	Nerve entrapment

	03Z	Other Soft tissue injury
	04	**Head Injury**
	04A	Concussion
	04B	Extradural Haemorrhage
	04C	Subdural Haemorrhage
	04D	Traumatic cerebral oedema
	04Z	Other head injury
	05	**Dental Injury**
	05A	Avulsion
	05B	Fracture
	05Z	Other dental injury
	06	**Vascular Injury**
	06A	Arterial haemorrhage
	06B	Arterial contusion
	06C	Arterial avulsion
	06D	Arterial perforation
	06E	Arterial rupture
	06F	Arterial transection
	06G	Venous haemorrhage
	06H	Venous contusion
	06J	Venous avulsion
	06K	Venous rupture
	06L	Venous transection
	06Z	Other vascula
	07	**Visceral injury**
	07A	Traumatic haemothorax
	07B	Traumatic pneumothorax
	07C	Pulmonary contusion
	07D	Traumatic haemopneumothorax
	07E	Traumatic haemopericardium
	07F	Cardiac contusion
	07G	Pneumomediastinum
	07H	Rupture *
	07J	Perforation *
	07K	Avulsion *
	08	**Burn**
	08A	1st degree/duperficial
	08B	2nd degree/partial thickness
	08C	3rd degree/full thickness
	09	**Scald**

	10	Corrosion
	10A	1st degree
	10B	2nd degree
	10C	3rd degree
	13	**Frostbite**
	13A	Superficial
	13B	With tissue necrosis
	13Z	Other frostbite
	14	**Poisoning**
	15	Electric shock
	16	Multiple injuries
	98	Other nature of injury
Diseases code		All ICD 10 mentioned in Appendix 1
Diagnosis code	01	Trauma/injury/poisoning
Procedure code	01	Wound care
	02	Burn care
	03	Limb immobilisation
	04A	Reduction of dislocation
	04B	Manipulation of fracture
	11A	Removal of foreign body from orifice
