# Supplementary material for: Behavioural difficulties in early childhood and risk of adolescent injury
Source: Arch Dis Child. 2019 Oct 30;105(3):282–7. doi: 10.1136/archdischild-2019-317271 (PMC7041499; doi:10.1136/archdischild-2019-317271)
Supplement: Supplementary data [file archdischild-2019-317271supp005.pdf]

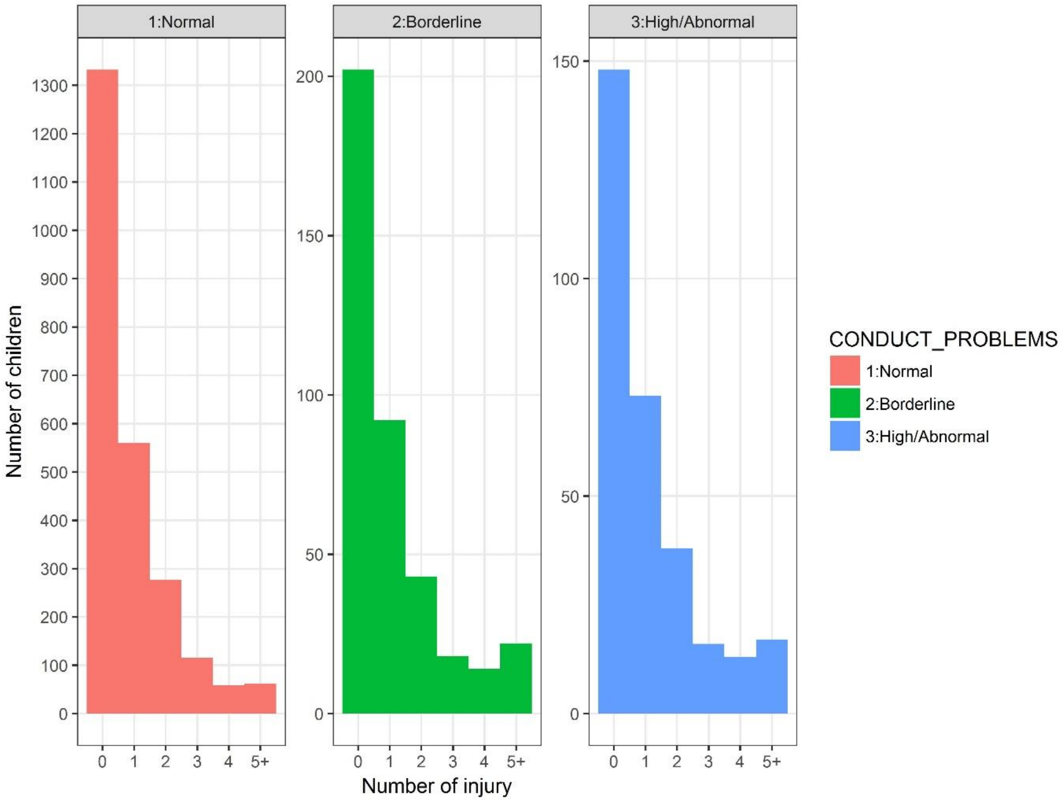

Figure 1: Distribution of number of injury across conduct problems categories. The mean and variances for each category were (0.87, 2.02); (1.10, 2.98); (1.16, 2.75) for Normal, Borderline and High/Abnormal.
