# Supplementary material for: Behavioural difficulties in early childhood and risk of adolescent injury
Source: Arch Dis Child. 2019 Oct 30;105(3):282–7. doi: 10.1136/archdischild-2019-317271 (PMC7041499; doi:10.1136/archdischild-2019-317271)
Supplement: Supplementary data [file archdischild-2019-317271supp004.pdf]

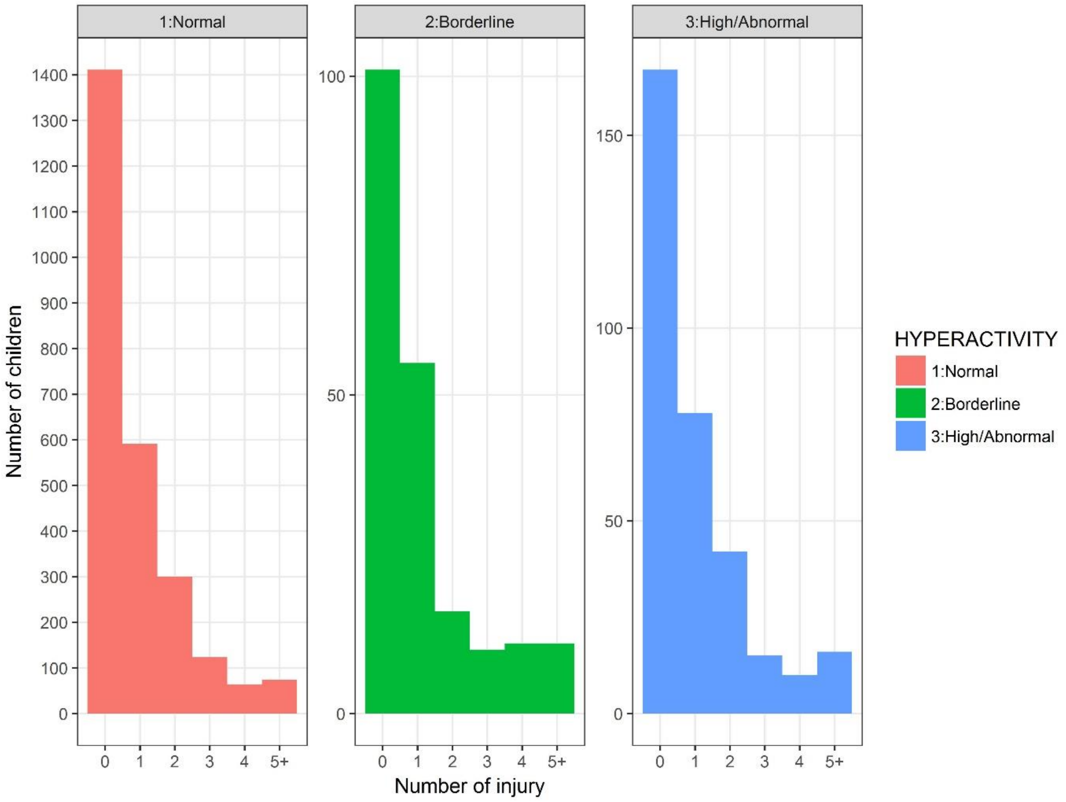

Figure 1: Distribution of number of injury across hyperactivity categories. The means and variances for each category were (0.09, 2.12); (1.15, 3.24); (1.04, 2.37) for Normal, Borderline and High/Abnormal
